# A Survey of Genomic Properties for the Detection of Regulatory Polymorphisms

**DOI:** 10.1371/journal.pcbi.0030106

**Published:** 2007-06-08

**Authors:** Stephen B Montgomery, Obi L Griffith, Johanna M Schuetz, Angela Brooks-Wilson, Steven J. M Jones

**Affiliations:** Canada's Michael Smith Genome Sciences Centre, British Columbia Cancer Agency, Vancouver, British Columbia, Canada; Weizmann Institute of Science, Israel

## Abstract

Advances in the computational identification of functional noncoding polymorphisms will aid in cataloging novel determinants of health and identifying genetic variants that explain human evolution. To date, however, the development and evaluation of such techniques has been limited by the availability of known regulatory polymorphisms. We have attempted to address this by assembling, from the literature, a computationally tractable set of regulatory polymorphisms within the ORegAnno database (http://www.oreganno.org). We have further used 104 regulatory single-nucleotide polymorphisms from this set and 951 polymorphisms of unknown function, from 2-kb and 152-bp noncoding upstream regions of genes, to investigate the discriminatory potential of 23 properties related to gene regulation and population genetics. Among the most important properties detected in this region are distance to transcription start site, local repetitive content, sequence conservation, minor and derived allele frequencies, and presence of a CpG island. We further used the entire set of properties to evaluate their collective performance in detecting regulatory polymorphisms. Using a 10-fold cross-validation approach, we were able to achieve a sensitivity and specificity of 0.82 and 0.71, respectively, and we show that this performance is strongly influenced by the distance to the transcription start site.

## Author Summary

Computational techniques are used in biology to prioritize DNA sequence variants (or polymorphisms) that may be responsible for population diversity and the manifestation of species-specific traits. Predominantly, they have been used to predict the class of polymorphisms that alter protein function through allele-specific changes to amino acid composition. However, polymorphisms that alter gene expression have been increasingly implicated in manifestation of similar traits. Prioritization of these polymorphisms is challenged, though, by the lack of knowledge regarding the mechanisms of gene regulation and the paucity of characterized regulatory polymorphisms. Our work attempts to address this issue by assembling a collection of regulatory polymorphisms from the existing literature. Furthermore, we use this collection to investigate and prioritize various properties that may be important for identifying novel regulatory polymorphisms.

## Introduction

Our ability to identify the molecular mechanisms responsible for specific genetic traits within our population will be enhanced by our imminent ability to decipher each individual's genome. This is evident from recent advances in sequencing and genotyping technologies, which allow an increasing number of variants to be sampled for association and linkage (reviewed in [1–3]) and contribute a growing number of sources of variation and their frequencies to public databases each year. As new variants are identified, each becomes a molecular window into our past, present, and future—each aids in tracing our genetic heritage and in charting the footsteps of our common evolution, and possesses the potential to predict disease or drug susceptibilities, ideally acting as an early-warning system in preventative medical practice (reviewed in [4,5]). However, our ability to catalog genotypes has far outstripped our ability to implicate them in phenotypes. Currently, more than 6 million unique single-nucleotide polymorphisms (SNPs) are included in version 126 of dbSNP [6]; of these SNPs, only a very small fraction have been associated with a phenotype using genetic association or linkage analysis. This is because association studies are costly, time-consuming, and dependent on the frequency of the genotype in the sampled population. Furthermore, many SNPs are not necessarily expected to have a function. To select candidates for functional validation, computational methods have been developed to identify SNPs that alter the protein-coding structure of genes [7–16]. These types of computational methods tend to prioritize putative functional SNPs by identifying those SNPs that alter a protein's amino acid sequence, are located within well-conserved regions or functional protein domains, and alter the biochemical structure of the protein. However, very few methods identify regulatory SNPs (rSNPs) that alter the expression of genes. Such rSNPs have been implicated in the etiology of several human diseases, including cancer [17,18], depression [19], systemic lupus erythematosus [20], perinatal HIV-1 transmission [21], and response to type 1 interferons [22]. This work aims to extend computer-based techniques to identify this particular class of functional variants within the core promoter regions of human genes.

Conventional computational approaches to rSNP classification have predominantly relied on allele-specific differences in the scoring of transcription factor weight matrices as supplied from databases such as TRANSFAC and Jaspar [15,16,23]. SNPs located within matrix positions possessing high information content are assumed more likely to be functional. Support for this hypothesis to date, however, has been restricted to single-case examples. Furthermore, a recent study has failed to detect significant weight matrix signals in 65% of regulatory polymorphisms (*n* = 40) [24]. However, the prevailing hypothesis in computational regulatory element prediction has been that the majority of predictions using unrestricted application of matrix-based approaches are false positives. By extending this technique and using phylogenetic footprinting between mouse and human, it was demonstrated that from ten SNPs that show significant allele-specific differences in Jaspar predictions, seven also demonstrated electrophoretic mobility shift differences [23]. However, only two of the seven had a marked effect in reporter gene assays. Conservation alone has also been demonstrated as a poor discriminant of function in a study of regulatory polymorphisms in Eukaryotic Promoter Database promoters, where zero of ten experimentally validated regulatory variants were in conserved binding sites [25].

A substantial challenge with developing strategies for identifying functional noncoding variants has been the shortage of characterized regulatory variants. Few studies have successfully identified the causative variant(s) after a susceptibility haplotype is identified. To address this problem, we have assembled the largest openly available collection of functional regulatory polymorphisms within the ORegAnno database (http://www.oreganno.org) [26]. From this dataset, we have examined several features of these SNPs as they relate to polymorphisms of unknown function (ufSNPs) within the promoter regions of associated genes (up to 2 kb). Our hypothesis is that using a combination of regulatory and population genetics properties, the discriminative efficacy of individual properties can be evaluated, and significant predictors of rSNP function can be chosen. Within our assayed set, we have found that the best discriminants are the distance to the transcription start site (TSS), local repetitive density and content, sequence conservation, minor allele frequency (MAF) and derived allele frequency, and CpG island presence. Notably, the unrestricted application of a matrix-based approach is demonstrated to be one of the least effective classifiers.

We have used this dataset of rSNPs and their properties to train a support vector machine (SVM) classifier. Two approaches were used to train the classifier: one in which the properties of all rSNPs were compared with that of all the ufSNPs, and one in which each property value of the positive SNPs and ufSNPs within an associated gene were compared with the average values for each property within that gene (referred to here as the “All” and “Group” approaches, respectively). The All approach is designed to determine if there are any properties that are important across the test set, while the Group approach is designed to determine if there are important directional shifts in values within a promoter that may discriminate functional SNPs from ufSNPs. In a 10-fold cross-validated test, the SVM achieves a receiver operating characteristic (ROC) value of 0.83 ± 0.05 for the All analysis (sensitivity, 0.82 ± 0.08; specificity, 0.71 ± 0.13) and 0.78 ± 0.04 for the Group analysis (sensitivity, 0.72 ± 0.19; specificity, 0.68 ± 0.07).

## Methods

### Data

Literature describing noncoding polymorphisms responsible for allele-specific differences in gene expression was surveyed from PubMed [27]. From this literature, 160 regulatory polymorphisms were identified in 103 publications; each was selected based on experimental evidence that confirmed its direct role in altering gene expression. This selection criterion specifically excluded those polymorphisms in which the experimental evidence could only confirm that the reported polymorphism was in linkage disequilibrium with an rSNP. Each identified rSNP was manually curated in the ORegAnno database. Subsequently, 104 polymorphisms were selected based on the criteria that they were SNPs (excluding seven insertion–deletion polymorphisms), and were within 2 kb of the TSS of their associated gene (as annotated in version 37 of EnsEMBL [28]; Table 1). A 2-kb region was chosen to maximize the number of rSNPs included while minimizing the size of sequence investigated; at 2 kb, the addition of a single further rSNP would increase the surveyed region by 43%, whereas the previous addition resulted in an increase of 9%. At this window size, 39 rSNPs were excluded from analysis. An additional ten polymorphisms were excluded because of deprecated annotation of the gene or discordant genomic location with the associated gene. In total, the remaining 104-rSNP set contained polymorphisms involved in altering the expression of 78 different transcripts.

**Table 1:**
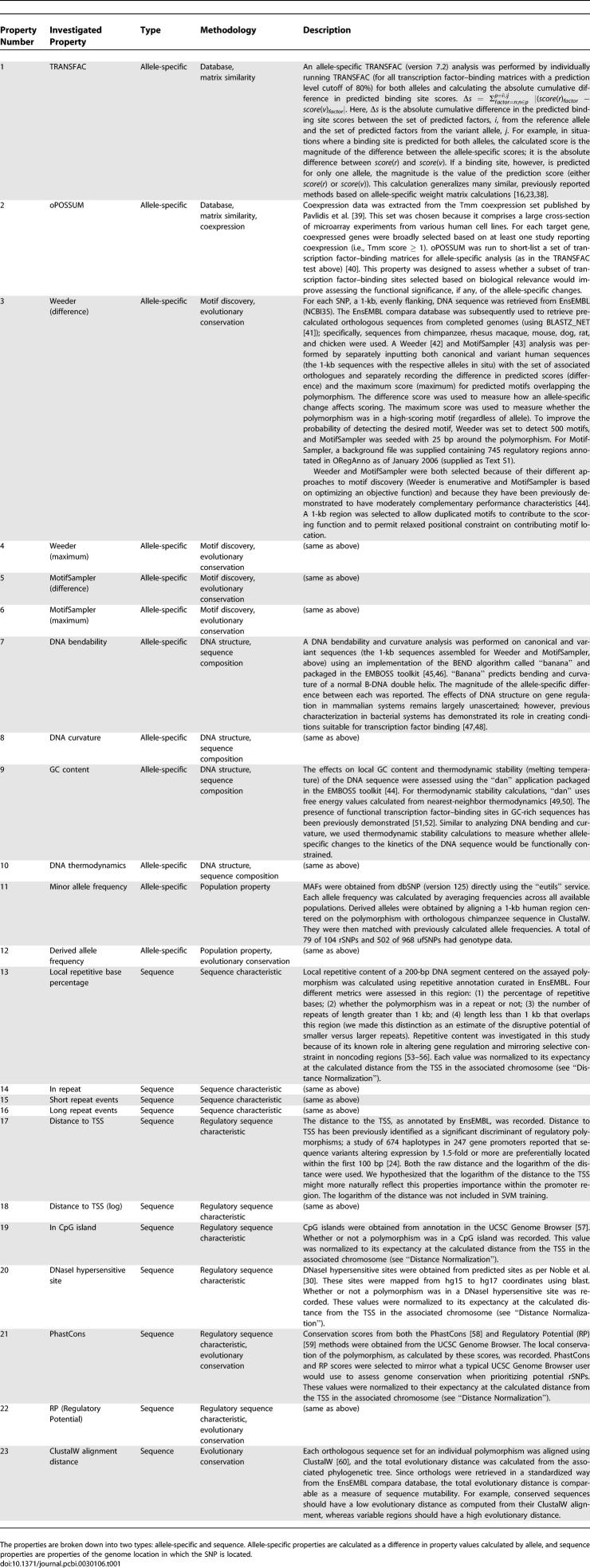
Investigated Properties

Using each of the 78 transcripts, SNPs within 2 kb of the TSS were extracted from version 37 of EnsEMBL (dbSNP version 125), producing exactly 951 ufSNPs. The ufSNP and rSNP genomic locations have been mapped (see Table S1).

### Investigated Properties

A total of 23 different properties of relevance to assessing regulatory function were calculated for each SNP in both the 104-rSNP and ufSNP sets (Table 1). These properties were selected to represent a cross-section of well-documented methodologies for assessing the functional significance of both allele-specific changes and DNA sequences within noncoding regions.

### Test Data Design (All and Group)

Two types of analyses were conducted using the investigated properties. One was an all-versus-all approach, where the 104-rSNP and ufSNP sets were compared en masse. The other was a group analysis, where the average value of each property within each upstream noncoding region was first calculated, and then the individual SNP properties within that region were recalculated as the difference from this average. The All test data were designed to identify global characteristics of rSNPs, while the Group test data were designed to look for directional trends within the sampled region that might be indicative of SNP importance. For example, the All test is able to ask whether rSNPs have generic features that would distinguish them from any other promoter SNP; the Group test is designed to identify whether there are any features that distinguish rSNPs from other SNPs within the same upstream noncoding region.

### SVM

The All and Group test data were input to the Gist SVM implementation [29]. We excluded the logarithmic distance to the TSS to prevent redundant classification with the raw distance to the TSS. Gist was run using the default parameters as described previously [30]. Of note, the Gist SVM requires that every value in the test and training parameter space is filled. To reflect the null hypothesis, that there are no differences between the ufSNPs and rSNPs, the All SVM was filled with promoter-specific average values wherever data could not be calculated. Likewise, the Group SVM was filled with zero values wherever data could not be calculated, indicating no divergence from average within the GROUP test set.

### Performance Measurement

The individual importance of each property in discriminating regulatory polymorphisms was assessed in the All and Group test sets using a Wilcoxon rank sum test. Each value was corrected for multiple testing using the BioConductor MTP package (http://www.bioconductor.org) by controlling for the family-wise error rate (α = 0.05 and B = 10,000) [31,32].

The performance of the Gist SVM classifier was measured using a ROC curve. ROC scores of 1 indicate perfect discrimination, while those at 0.5 indicate random classification of the input SNPs. ROC performance measurements have been previously described in detail elsewhere [30].

A 10-fold cross-validation was performed to assess the overall performance of the SVM. The input data was randomly partitioned by transcript into ten sets. Data from one set were excluded, and the remaining nine sets were trained on for each fold validation. This analysis was performed for each set to cover the entire training site and to calculate an average ROC value for the SVM.

### Distance Normalization

**Figure 1:**
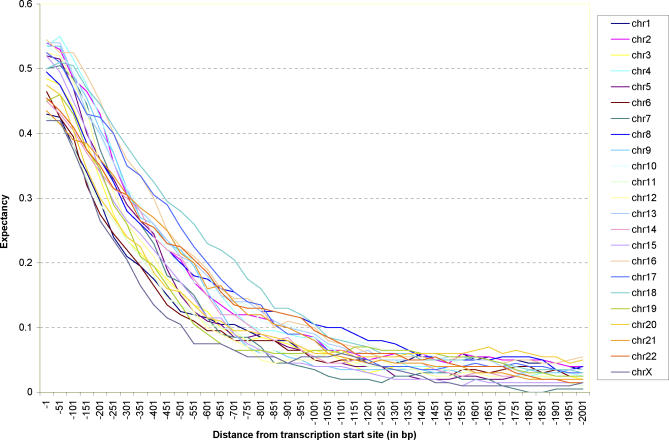
CpG Island Positional Bias CpG island expectancy is plotted for each chromosome as a function of the distance from the TSS. This type of data was used to normalize many of the features in this study for distance from the TSS. In this figure, the expectancy of being in a CpG island at position −1 for any promoter region is ~0.5.

We were concerned that several properties may be indirect measurements of distance from the TSS, and that any discrimination strategy would be limited to characterizing this property alone. This concern is a particular challenge since distance ascertainment bias exists; most SNPs surveyed were within a few hundred base pairs of the TSS, which is much smaller when compared with our sampling distance of 2 kb. Furthermore, it has been well established in a previous study that distance to the TSS is correlated to detection of rSNPs (it is unknown if this is because they are more likely to affect essential transcription factor–binding sites, or because there is a higher density of transcription factor–binding sites in these regions) [24]. For this reason, the discrimination potential of distance to the TSS could not be ignored. To adjust for bias, however, we calculated the expectancy of observing a feature at a particular distance from the TSS for each individual chromosome (Figure 1; CpG islands are shown as an example of this trend). This expectancy value was used to normalize the observation values for several of the properties in this study (identified in Table 1). This was performed by subtracting the expectancy value from the observed value. The impact of this normalization is negligible when comparing normalized ROC values against unnormalized ROC values; using a 10-fold cross-validation, the unnormalized ROC values for the ALL test are 0.82 ± 0.05 (unnormalized) and 0.83 ± 0.05 (normalized), and values for the GROUP test are 0.79 ± 0.04 (unnormalized) compared with 0.78 ± 0.07 (normalized).

## Results

### Property Ranking

A total of 104 rSNPs and 951 ufSNPs in the upstream noncoding regions of 78 genes were compiled to test properties that discriminate polymorphisms with effects on gene expression. A multiple testing–corrected Wilcoxon rank sum test was used to analyze the All test data (Table 2). Analyzing the All test data identified several properties of significance in discriminating between rSNPs and ufSNPs (*p* < 0.05). The properties of significance in the All test data, in order of importance, were: 1) distance to the TSS (properties 13 and 14); 2) in a CpG island (property 19); 3) long repeat events (property 16); 4) local repetitive base percentage (property 13); 5) derived allele frequency (property 12); 6) minor allele frequency (MAF; property 11); 7) Regulatory Potential score (property 22); 8) in a repeat (property 14); and 9) ClustalW alignment distance (property 23).

**Table 2:**
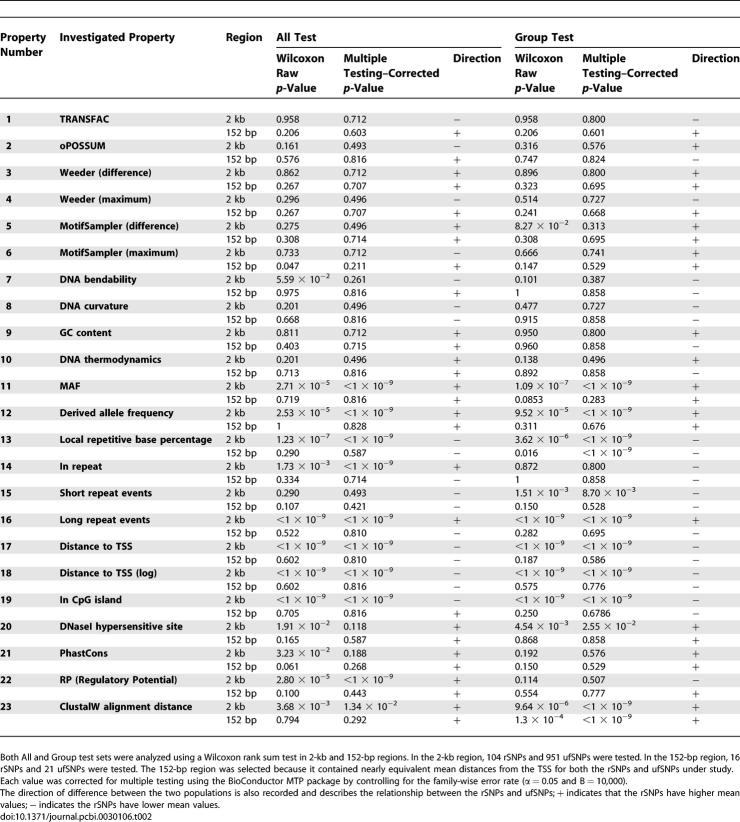
Analysis of rSNP and ufSNP Properties in the 2-kb and 152-bp Upstream Noncoding Regions

However, a concern with the All analysis was that calculated property values for SNPs in individual upstream noncoding regions would not be comparable with those in other upstream noncoding regions due to differences in background property values. To address this, a multiple testing–corrected Wilcoxon rank sum test was also used to analyze the Group test data (Table 2). The properties of significance (*p* < 0.05) in the Group test data, in order of importance, were: 1) distance to the TSS (properties 13 and 14); 2) long repeat events (property 16); 3) in a CpG island (property 19); 4) MAF (property 11); 5) local repetitive base percentage (property 13); 6) ClustalW alignment distance (property 23); 7) derived allele frequency (property 12); 8) short repeat events (property 15); and 9) DNaseI hypersensitive site (property 20).

Both lists are highly concordant and demonstrate several properties that may be of utility when prioritizing SNPs for functional analysis either across the genome or within an individual upstream noncoding region. In both tests, distance to the TSS was found to be the most significant discriminant. While it is possible that ascertainment bias in the 104-rSNP set contributes to the strength of this discriminant in our study, this property has also been independently identified as an important discriminant in a previous study where, in 500-bp assayed regions, 50% of rSNPs identified through transfection experiments were within 100 bp of the TSS (*n* = 40) [24].

Furthermore, several other properties are consistently identified as being significant after normalization against distance to TSS. One property, ClustalW alignment distance, was identified in both the All and Group tests as being significant. The mean value of ClustalW alignment distance was slightly higher for the tested rSNPs compared with the ufSNPs, indicating that 1-kb multiple alignments centered on the tested rSNPs were more divergent than those centered on ufSNPs. This result is concordant with previous analyses of conservation around rSNPs (*n* = 10) [25]. However, trends in the other conservation scores used in this study, while nonsignificant in discriminating between the tested rSNP and ufSNPs, conversely suggest that the tested rSNPs are more conserved than ufSNPs. Since these metrics use tighter window sizes than those used for calculating the ClustalW alignment distance, this result suggests that increased mutation around an rSNP may be more informative than the conservation status of the rSNP itself.

Another property of significance was repetitive element content. Our results indicate that the tested rSNPs were less likely to be in or around repetitive elements. This suggests that regions that are likely to contain a transcription factor–binding site are less likely to accrue repetitive elements and be subject to dysregulation. We note, however, that ascertainment bias by which the 104-rSNPs set was surveyed in terms of repetitive elements is not known, and future collections of discovered rSNPs should address this issue.

Both MAF and derived allele frequency are also identified as significant discriminants. Unexpectedly, for genotyped SNPs, the MAF was higher in the 104-rSNP set than in the ufSNP set. Previous analyses of MAF have suggested that most functional SNPs are positioned around 6% [33] or possess no allele frequency bias [24]. In this study, the average MAF was approximately 22%. Since a subset of the 104-rSNP set has been derived from association studies, it is possible that ascertainment bias may explain part of this result as researchers may preferentially be choosing higher MAF SNPs because of their greater statistical power. Of further interest, the derived allele frequency was higher in the 104-rSNP set than in the ufSNP set. This could suggest that many of the derived alleles have been driven to higher frequencies due to new variants increasing in frequency in our population, through either population bottlenecks or positive selection. The former hypothesis is supported by the supplemental observation that when restricting populations to HapMap (http://www.hapmap.org) phase I populations only, the Asian and European populations mirror this result, while the African population has lower MAFs on average. The latter hypothesis, however, supports a model of evolution of genetic susceptibility to common diseases explained by ancient alleles recently becoming predisposed to disease due to changes in human lifestyle and life expectancy [34].

Another interesting result was that SNPs in the 104-rSNP set were less likely to be in CpG islands than were ufSNPs. Since CpG expectancy was normalized from average values at specific distances from the TSS of associated genes across individual chromosomes, an admixture of CpG and CpG-less promoters would drive the 104-rSNP set values lower than the ufSNP set values (Figure 1) [35,36]. However, without normalization, the significance of this value for the All and Group tests is similar (All, *p* = 3.78 × 10^−5^; Group, *p* = 1.96 × 10^−3^), suggesting that the rSNPs are in fact less likely to be in CpG islands.

Many tested properties fell below our significance threshold in these tests. Of interest, both weight matrix–based approaches did not discriminate well. In addition, our definition of coexpression was significantly broad as to allow multiple coexpressed partners for any given gene; this may have reduced the overall effectiveness of reducing transcription factor–binding profiles using this information. However, the performance of the coexpression-filtered approach was moderately better than the TRANSFAC approach alone. This suggests that targeted analysis of specific, biologically relevant transcription factors may further increase the discriminating ability of this approach. This should also act as a warning to those who have in the past applied the TRANSFAC approach to this problem indiscriminately. Furthermore, none of the DNA structural or stability analyses used were successfully discriminatory. This analysis could indicate that not only do these features have nongeneralizable effects using the data in this study, but since these analyses also measure local sequence composition, no particularly important effect is caused by specific base changes.

### SVM Cross-Validation

**Figure 2:**
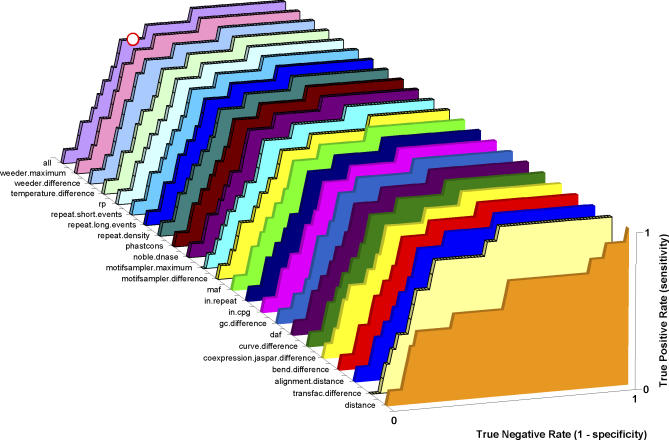
ROC Curves for Discriminating Known rSNPs from ufSNPs Representative ROC curves were calculated by training an SVM on a 90% subset of the 104-rSNP and ufSNP datasets. Here, 93 rSNPs and 882 ufSNPs were used for training, followed by testing on the held-out 10%. The ALL SVM approach was used for training. Furthermore, each curve had one tested property held out to demonstrate the impact of various properties on training. Notably, many curves are the same except for a marked reduction in performance when the “Distance to TSS” property is held out. The area under the “all” curve is 0.830. The dot on the “all” curve marks the location of the decision boundary selected by the SVM. At this boundary, the SVM identifies nine of 11 true positives and 56 of 69 true negatives. (Plots for each tested partition are available at http://www.bcgsc.ca/chum/gistplots.html).

To evaluate whether the combination of the tested properties would enhance discrimination of rSNPs from ufSNPs, we trained a SVM for the ALL and GROUP test data. We tested the classification performance of SVMs by 10-fold cross-validation. For each SVM, the mean area under the ROC curve was 0.83 ± 0.05 and 0.78 ± 0.04, respectively. Both suggest good performance. It is significant, however, that when removing distance from the classification, the performance of each test drops to 0.52 ± 0.09 and 0.48 ± 0.07, respectively (Figure 2). This reduction in performance should not be taken to indicate that other properties identified in the multiple testing–corrected Wilcoxon rank sum test are not actually discriminatory since 10-fold cross-validation of All and Group test SVMs built with only the properties identified as significant using the multiple testing–corrected Wilcoxon rank sum test (*p* < 0.05) and excluding distance to the TSS achieved ROC values of 0.77 ± 0.08 and 0.75 ± 0.07, respectively. This result suggests that nonsignificant results may act to overparameterize the SVM model and mask subtle, true discriminatory signals.

### Distance Analysis

To address the issue of distance bias further, we fortuitously identified that, across our dataset, in the 152 bp immediately upstream of the TSS, the average distance to the TSS for the ufSNPs was identical to that of the rSNPs. This 152-bp window therefore represented a region with no observable distance biases, albeit a greatly reduced subset in size; at this window size, only 16 rSNPs and 21 ufSNPs were available for analysis. When analyzed using a multiple testing–corrected Wilcoxon rank sum test for both All and Group test sets, only two properties were significant (*p* < 0.05): repetitive element density (property 13) and ClustalW alignment distance (property 23) (Table 2). We further tested window sizes of 500 bp, 1 kb, and 1.5 kb and noticed only a gradual reduction in performance of the tested properties for smaller window sizes (see Table S2).

**Figure 3:**
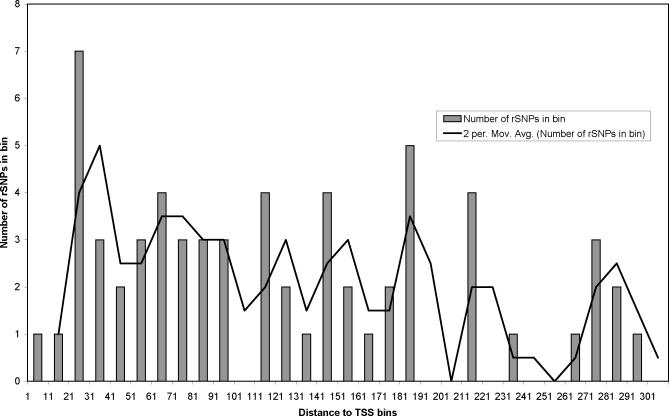
Histogram of Positional Bias of rSNPs for the First 300 bp of Sequence The positions of rSNPs are plotted in a histogram for bin sizes of 10 bp for the first 300 bp of sequence from the TSS. A blip is seen at position 21–31, where it is likely that TATA and CCAAT box–binding sites are located. These types of rSNPs, however, are only slightly overrepresented in this study and from this graph are not expected to significantly bias the outcome.

We also examined the position of identified rSNPs to characterize possible bias. Our expectation was that well-established transcription factor–binding sites such as the TATA and CCAAT boxes may be overrepresented and contribute to lower distance values. A histogram of rSNPs for the first 300 bp of sequence from the TSS shows an expected increase around the 21–31 position where seven rSNPs are located, twice as many as average. However, it is apparent that these types of binding sites are only overrepresented slightly when compared with the distribution of rSNPs at other positions (Figure 3).

### Availability

All pipeline software has been programmed in Perl and is available under the Lesser GNU Public Licence at http://www.bcgsc.ca/chum under the name CHuM (*cis*-acting human mutation modules). All data are available from this site.

## Discussion

This study introduces the largest publicly available collection of rSNPs—160 known rSNPs from literature. Using this collection, we investigate 104 rSNPs and 951 ufSNPs in human 2-kb upstream regions to identify properties that may discriminate functional from nonfunctional polymorphisms. We identify several properties that may be useful to researchers attempting to determine the functional status of upstream noncoding SNPs. The most important properties detected suggest that rSNPs are close to the TSS, are not within CpG islands, are isolated from repetitive elements, possess higher MAF and higher derived allele frequency, and are within comparatively more divergent regions. However, within a 152-bp window, where an equal distribution of rSNPs and ufSNPs from the TSS is obtained, the significant results suggest that only repetitive element content and local divergence remain important (we have included in Table S2 information on how property significance changes with window size). We further combined each of the properties identified in the 2-kb region to train an SVM to classify the functional status of the 104-rSNP set and 951-ufSNP set. We hypothesized that subtle differences in individual properties may be more important than any one property in detecting rSNPs. It is of note, despite mentioned ascertainment biases, that our sensitivity and specificity for the All test was 0.82 ± 0.08 and 0.71 ± 0.13, respectively, and for the Group test was 0.72 ± 0.19 and 0.68 ± 0.07, respectively. Also of note, the strength of the distance to the TSS as a discriminatory property was demonstrated in both tests when removal of the property significantly reduced the effectiveness of the classifier to near random performance. However, we observed that this reduction in performance was recovered in part when only the properties identified as significant through the multiple testing–corrected Wilcoxon rank sum test (*p* < 0.05) in the 2-kb All and Group tests were applied, and the distance to the TSS was excluded.

Through this work, several challenges are apparent with current predictive approaches to prioritize candidate rSNPs. Necessary to future analyses is a dataset of core promoter polymorphisms that are nonfunctional across a broad range of cell types; since our negative control set was a neutral set, it is assured that more accurate performance metrics can come from addition of a reliable negative control set. Furthermore, recent analysis of allelic expression difference has demonstrated that the effects of rSNPs may be highly context-specific such that function in one cell line may not imply function in others; to address this complication, future analysis may require expanded collections of cell line–specific positive and negative rSNPs [37]. Future studies of promoter polymorphisms will also need to take advantage of known transcription factor–binding sites. Such information will be invaluable in dissecting the causal nature of many of the properties.

In summary, this study introduces a new dataset for the investigation of rSNPs. We have also introduced one of the first gene regulation and population genetics–based approaches to classifying rSNPs in the core promoter regions of human genes. We identify the utility of different gene regulation and population genetics properties in discriminating literature-curated rSNPs. Such results are increasingly essential to researchers seeking criteria for prioritizing SNPs to test in association, binding, or expression assays. Furthermore, we provided evidence that popular methodological practices based on identification of allele-specific differences in position weight matrices through unrestricted application of the TRANSFAC database are poor criteria for SNP selection. However, we highlight the fact that because of the lack of extensive unbiased collections of rSNPs, it still remains challenging to dissect the existing effects of investigator or methodological biases in evaluating the importance of these properties. We hope that this work will stimulate active discussion and both the development of expanded collections of rSNPs and an improved class of bioinformatics tools for rSNP analysis that address these challenges.

## Supporting Information

Figure S1 Mapped rSNPs and ufSNPs The locations of the tested rSNPs and ufSNPs are plotted upstream of their respective genes.
(6.4 MB PNG)

Table S1 Tested rSNPs The tested rSNP data is listed with information describing experimental evidence, associated gene, and dbSNP number, if available.
(59 KB PDF)

Table S2 Performance of Genomic Properties at 500-bp, 1,000-bp, and 1,500-bp Window Sizes Different upstream window sizes were selected for All and Group analyses. The results of the Wilcoxon rank sum test for these windows are summarized and displayed as figures.
(44 KB XLS)

Text S1 Background File for MotifSampler Promoters annotated in ORegAnno were assembled into this background file for MotifSampler analysis.
(5 KB RTF)

## Abbreviations

MAF: minor allele frequency
ROC: receiver operating characteristic
rSNP: regulatory single-nucleotide polymorphism
SVM: support vector machine
TSS: transcription start site
ufSNP: SNP of unknown function
