# Supplementary material for: A Survey of Genomic Properties for the Detection of Regulatory Polymorphisms
Source: PLoS Comput Biol. 2007 Jun 8;3(6):e106. doi: 10.1371/journal.pcbi.0030106 (PMC1892352; doi:10.1371/journal.pcbi.0030106)
Supplement: Supplementary file 1 [file pcbi.0030106.st001.pdf]

		ORegAnno Supporting Evidence Classes											
**Gene Name** (Entrez Gene ID / EnsEMBL Stable Transcript ID)	**ORegAnno Stable ID** (dbSNP ID)	**OREGES00001** Direct Gel Shift	**OREGES00002** Supershift	**OREGES00003** Gel Shift Competition	**OREGES00004** Transient Transfection Luciferase Assay	**OREGES00007** RT-PCR	**OREGES00009** Allele-specific Transcript Quantification	**OREGES00010** Resequencing	**OREGES00011** Single-stranded Conformational Polymorphism	**OREGES00015** DNase Footprinting Assay	**OREGES00019** CAT Assay	**OREGES00020** RLM-RACE	**OREGES00033** Methylation Interference Assay
ABCA1<br>EntrezGene:19<br>EnsEMBL:ENST00000297693	OREG0001970<br>rs2422493			2	1	1							
ADH4<br>EntrezGene:127<br>EnsEMBL:ENST00000265512	OREG0000364<br>rs1800759				1								
ADRB2<br>EntrezGene:154<br>EnsEMBL:ENST00000305988	OREG0000076<br>rs11959427	1					1						
AGER<br>EntrezGene:177<br>EnsEMBL:ENST00000326486	OREG0001961<br>rs1800625	2			1								
	OREG0001962<br>rs1800624	2		1	1								
AGRP<br>EntrezGene:181<br>EnsEMBL:ENST00000290953	OREG0001960<br>rs5030981	2			2								
AGT<br>EntrezGene:183<br>EnsEMBL:ENST00000258224	OREG0000409<br>rs5050	1	2								4		
	OREG0001957<br>rs1800585			1	1								
	OREG0001958<br>rs5051	1		1							2		
AKR1B1<br>EntrezGene:231<br>EnsEMBL:ENST00000285930	OREG0000093	1			1								
APOA1	OREG0001956			2	1						1		

EntrezGene:335<br>EnsEMBL:ENST00000236850	rs17243116												
APOA2<br>EntrezGene:336<br>EnsEMBL:ENST00000289906	OREG0001857				2								
	OREG0001955	1		1							1		
APOB<br>EntrezGene:338<br>EnsEMBL:ENST00000233242	OREG0001954<br>rs934197	1		1							1		
APOC3<br>EntrezGene:345<br>EnsEMBL:ENST00000227667	OREG0001952	1									1		
	OREG0001953			1	1								
APOE<br>EntrezGene:348<br>EnsEMBL:ENST00000252486	OREG0001950<br>rs449647	1			1								
	OREG0001951<br>rs405509	1			1								
APOH<br>EntrezGene:350<br>EnsEMBL:ENST00000205948	OREG0000089<br>rs8178822	1			1								
APP<br>EntrezGene:351<br>EnsEMBL:ENST00000346798	OREG0001905<br>rs466448	1		1							1		
CALM1<br>EntrezGene:801<br>EnsEMBL:ENST00000356978	OREG0000146<br>rs12885713	1			1	1		1					
CAT<br>EntrezGene:847<br>EnsEMBL:ENST00000241052	OREG0001834<br>rs17880664				2								
	OREG0001949<br>rs17879971	2		1	2								
CCL5<br>EntrezGene:6352<br>EnsEMBL:ENST00000293272	OREG0000007<br>rs2107538		1		1				1				
	OREG0001920<br>rs2280788				2			1					
CD14<br>EntrezGene:929<br>EnsEMBL:ENST00000302014	OREG0000090<br>rs2569190		1	1	1								
CDH1<br>EntrezGene:999<br>EnsEMBL:ENST00000261769	OREG0001948<br>rs16260	1		1	1					1			
CETP<br>EntrezGene:1071	OREG0001947<br>rs1800775	1	1	1	2				1				

EnsEMBL:ENST00000200676													
CFTR EntrezGene:1080 EnsEMBL:ENST00000003084	OREG0000094	1	1	1	1								
COL18A1 EntrezGene:80781 EnsEMBL:ENST00000359759	OREG0001732 rs755548	1		1									
CRYAA EntrezGene:1409 EnsEMBL:ENST00000291554	OREG0001861				2								
CSDA EntrezGene:8531 EnsEMBL:ENST00000228251	OREG0000014				1								
CTLA4 EntrezGene:1493 EnsEMBL:ENST00000302823	OREG0000107 rs5742909				1								
CYP2A6 EntrezGene:1548 EnsEMBL:ENST00000301141	OREG0000012 rs4803381	1			1								
CYP2B6 EntrezGene:1576 EnsEMBL:ENST00000324071	OREG0000102	2			1							1	
CYP3A4 EntrezGene:1555 EnsEMBL:ENST00000336411	OREG0000071	1			1								
	OREG0000099	1						1					
DLD EntrezGene:1738 EnsEMBL:ENST00000205402	OREG0001837 rs6943999				2								
DNASE2 EntrezGene:1777 EnsEMBL:ENST00000222219	OREG0001946				2								
DRD4 EntrezGene:1815 EnsEMBL:ENST00000176183	OREG0001945 rs12720380										1		
ECE1 EntrezGene:1889 EnsEMBL:ENST00000264205	OREG0001971 rs213045	1	1		1								
ERCC1 EntrezGene:2067 EnsEMBL:ENST00000300853	OREG0001838 rs3212931				2								

F7<br>EntrezGene:2155<br>EnsEMBL:ENST00000261967	OREG0000406			1	1								
	OREG0001941<br>rs510317	1		1							1		
	OREG0001942<br>rs7981123	1		1							1		
FECH<br>EntrezGene:2235<br>EnsEMBL:ENST00000262093	OREG0000101	1	1		1								
FGB<br>EntrezGene:2244<br>EnsEMBL:ENST00000302068	OREG0001939<br>rs1800791	1									1		
	OREG0001940<br>rs1800790	1			1								
GFPT1<br>EntrezGene:2673<br>EnsEMBL:ENST00000357308	OREG0000073				1								
GSTA1<br>EntrezGene:2938<br>EnsEMBL:ENST00000334575	OREG0001845<br>rs11964968				2								
	OREG0001846				2								
HBG2<br>EntrezGene:3048<br>EnsEMBL:ENST00000336906	OREG0000405	1		1						1			
	OREG0000411	1	1										
HLA-DQB1<br>EntrezGene:3119<br>EnsEMBL:ENST00000334399	OREG0001969<br>rs11568829			1							1		
HTR2C<br>EntrezGene:3358<br>EnsEMBL:ENST00000276198	OREG0001848<br>rs3813929				2								
IAPP<br>EntrezGene:3375<br>EnsEMBL:ENST00000240652	OREG0000095<br>rs11836625	1	1	1	1								
IGFBP3<br>EntrezGene:3486<br>EnsEMBL:ENST00000275521	OREG0001936<br>rs2854744	1			1								
IL6<br>EntrezGene:3569<br>EnsEMBL:ENST00000258743	OREG0000015<br>rs1800795				1								
ITGA2<br>EntrezGene:3673<br>EnsEMBL:ENST00000296585	OREG0001934<br>rs28095	3	1		1								
	OREG0001935	1	1		1					1			

	rs27646												
KIAA0513<br>EntrezGene:9764<br>EnsEMBL:ENST00000258180	OREG0001831				2								
	OREG0001832				2								
LCT<br>EntrezGene:3938<br>EnsEMBL:ENST00000264162	OREG0000096	1											
LMO4<br>EntrezGene:8543<br>EnsEMBL:ENST00000271199	OREG0001863<br>rs3806174				2								
LPL<br>EntrezGene:4023<br>EnsEMBL:ENST00000311322	OREG0001933<br>rs1800590	1	1	1	8								
MGP<br>EntrezGene:4256<br>EnsEMBL:ENST00000228938	OREG0001932<br>rs1800802	1	1		2				1				
MMP12<br>EntrezGene:4321<br>EnsEMBL:ENST00000326227	OREG0001930<br>rs2276109	1	1	1	2				1				
MMP2<br>EntrezGene:4313<br>EnsEMBL:ENST00000219070	OREG0001899<br>rs243865	3	1	1	3								
	OREG0001900				1								
MMP9<br>EntrezGene:4318<br>EnsEMBL:ENST00000216953	OREG0001931<br>rs3918242	3			1				1		1		1
MPO<br>EntrezGene:4353<br>EnsEMBL:ENST00000225275	OREG0001887<br>rs2333227										1		
MSMB<br>EntrezGene:4477<br>EnsEMBL:ENST00000358559	OREG0001839<br>rs10993994				2								
MYOC<br>EntrezGene:4653<br>EnsEMBL:ENST00000037502	OREG0001836				2								
NCAM2<br>EntrezGene:4685<br>EnsEMBL:ENST00000284894	OREG0001859<br>rs3737412				2								
	OREG0001860				2								
NEFL<br>EntrezGene:4747	OREG0001862				2								

EnsEMBL:ENST00000311854													
NPY<br>EntrezGene:4852<br>EnsEMBL:ENST00000242152	OREG0001851<br>rs16147				2								
NPY2R<br>EntrezGene:4887<br>EnsEMBL:ENST00000329476	OREG0001849				2								
PCSK1<br>EntrezGene:5122<br>EnsEMBL:ENST00000311106	OREG0001872				2								
PON1<br>EntrezGene:5444<br>EnsEMBL:ENST00000222381	OREG0001925<br>rs705379				1								
	OREG0001926<br>rs705381				1								
	OREG0001927<br>rs854572				1								
	OREG0001928<br>rs854571				1								
PRM2<br>EntrezGene:5620<br>EnsEMBL:ENST00000241808	OREG0001828				2								
	OREG0001829				2								
PROC<br>EntrezGene:5624<br>EnsEMBL:ENST00000234071	OREG0000108	1	1	1	1								
PRODH<br>EntrezGene:5625<br>EnsEMBL:ENST00000334029	OREG0001847<br>rs386049				2								
PTGS2<br>EntrezGene:5743<br>EnsEMBL:ENST00000186982	OREG0000105<br>rs20417				1				1				
PTPRCAP<br>EntrezGene:5790<br>EnsEMBL:ENST00000326294	OREG0001835<br>rs869736				2								
RETN<br>EntrezGene:56729<br>EnsEMBL:ENST00000221515	OREG0000075<br>rs1862513		1	1	1			1					
SERPINA3<br>EntrezGene:12<br>EnsEMBL:ENST00000261981	OREG0001923<br>rs1884082				2								

SERPIND1<br>EntrezGene:3053<br>EnsEMBL:ENST00000215727	OREG0001866<br>rs165730				2								
SLC6A4<br>EntrezGene:6532<br>EnsEMBL:ENST00000261707	OREG0000313<br>rs25531	1						1					
SLC9A1<br>EntrezGene:6548<br>EnsEMBL:ENST00000263980	OREG0001827<br>rs11588974				2								
SPP1<br>EntrezGene:6696<br>EnsEMBL:ENST00000237623	OREG0000088	2			1								
	OREG0000104	1	1		1								
TBX21<br>EntrezGene:30009<br>EnsEMBL:ENST00000177694	OREG0000100<br>rs4794067	1			1			1					
TCOF1<br>EntrezGene:6949<br>EnsEMBL:ENST00000323668	OREG0000314	2			1								
TF<br>EntrezGene:7018<br>EnsEMBL:ENST00000264998	OREG0001868<br>rs4481157				2								
	OREG0001869<br>rs8177186				2								
THBD<br>EntrezGene:7056<br>EnsEMBL:ENST00000317606	OREG0001959<br>rs13306848				1				1				
TNF<br>EntrezGene:7124<br>EnsEMBL:ENST00000229681	OREG0000106<br>rs1800629	1			1								
	OREG0000110<br>rs1800750	1	1	1	1					1			
	OREG0001903<br>rs1800630	2		1	1								

Table 1: Investigated 104-rSNPs. Each rSNP appears as an ORegAnno entry and dbSNP entry (if available) along with information describing the evidence that was used to identify this rSNP in its original publication. Each listed rSNP occurs within 2kb upstream of the EnsEMBL-annotated transcription start site (EnsEMBL transcript ids and associated EntrezGene ids are provided).
