# Supplementary material for: A Survey of Genomic Properties for the Detection of Regulatory Polymorphisms
Source: PLoS Comput Biol. 2007 Jun 8;3(6):e106. doi: 10.1371/journal.pcbi.0030106 (PMC1892352; doi:10.1371/journal.pcbi.0030106)
Supplement: Supplementary file 2 [file pcbi.0030106.sd001.rtf]

#INCLUSive Background Model v1.0
#
#Order = 1
#Organism = human
#Sequences = human_promoters.fa
#Path = 
#

#snf
0.238204	0.256902	0.262515	0.242378	

#oligo frequency
0.238204
0.256902
0.262515
0.242378

#transition matrix
0.280865	0.201054	0.314597	0.203485	
0.273777	0.313409	0.123562	0.289252	
0.235542	0.257769	0.314527	0.192161	
0.161058	0.251125	0.302436	0.285381
